# Targeting Natural Killer Cells for Improved Immunity and Control of the Adaptive Immune Response

**DOI:** 10.3389/fcimb.2020.00231

**Published:** 2020-05-19

**Authors:** Stephen Pierce, Eric S. Geanes, Todd Bradley

**Affiliations:** ^1^Center for Pediatric Genomic Medicine, Children's Mercy Kansas City, Kansas City, MO, United States; ^2^Departments of Pediatrics and Pathology and Laboratory Medicine, University of Kansas Medical Center, Kansas City, KS, United States; ^3^Department of Pediatrics, University of Missouri Kansas City Medical School, Kansas City, MO, United States

**Keywords:** natural killer (Nk) cell, adaptive immune cells, NK cell therapy, vaccine, innate and adaptive immune response

## Abstract

Natural killer (NK) cells are critical for targeting and killing tumor, virus-infected and stressed cells as a member of the innate immune system. Recently, NK cells have also emerged as key regulators of adaptive immunity and have become a prominent therapeutic target for cancer immunotherapy and infection control. NK cells display a diverse array of phenotypes and function. Determining how NK cells develop and are regulated is critical for understanding their role in both innate and adaptive immunity. In this review we discuss current research approaches into NK cell adaptive immunity and how these cells are being harnessed for improving cancer and vaccination outcomes.

## NK Cell Cells Are Innate Immune Killers

Natural Killer (NK) cells are cytotoxic granular lymphoid cells that develop from a common progenitor of B and T cells (Kondo et al., 1997; Abel et al., 2018). NK cells have the innate ability to recognize both virally infected and tumor cells, play a key role in tumor clearing (Rosenau and Moon, 1961; Smith, 1966; Herberman et al., 1975; Kiessling et al., 1975; Yang et al., 2006), and the primary immunological response to viral infection (Biron et al., 1999; Vidal et al., 2011). When cytotoxic NK cells are activated, they release cytolytic granules and secrete inflammatory cytokines and chemokines that activate and recruit components of both the innate and adaptive immune response (Iannello and Raulet, 2013).

NK cell activation is governed by the ligand-receptor interactions of the activating and inhibitory receptors expressed on the NK cell surface (Tassi et al., 2006; Lanier, 2008; Bryceson et al., 2011). The balance of activating and inhibitory signals controls NK cell activation and function (MacFarlane and Campbell, 2006). NK cell activating receptors have well documented interactions with both viral (Alsheikhly et al., 1985; Mandelboim et al., 2001; Jarahian et al., 2009) and tumor derived ligands (Sivori et al., 1997; Vitale et al., 1998; Pende et al., 1999). NK cells also have killer cell immunoglobulin-like receptors (KIRs) that are vital to the normal function of NK cells and are critical for the education of NK cells. It is through these receptors that NK cells learn tolerance of self through HLA-I molecules, which serve as ligands to inhibitory KIRs (Ljunggren and Karre, 1990; Campbell and Purdy, 2011). Diversity of KIR genotypes among individuals that contribute to KIR-HLA interactions have implications for NK cell function and response against tumors and viruses.

## NK Cells With Adaptive Immune Cell Properties

Classically, NK cells are regarded as members of the innate immune system, but recent studies have elucidated that NK cells can display both adaptive and memory-like phenotypes. Antigen-specific NK cell memory was first described in T and B cell deficient mice displaying hapten-specific contact hypersensitivity (CHS) in skin cells after adoptive transfer of NK cells from a previously sensitized donor (O'Leary et al., 2006).

In addition to NK cell memory against haptens, it was discovered that murine NK cell receptor Ly49H showed specificity for MCMV-derived m157 expressed in mice (Daniels et al., 2001; Lee et al., 2001; Arase et al., 2002). This interaction between host Ly49H and virally-derived m157 elicits the clonal expansion of MCMV-activated NK cells, as well as the persistence of memory NK cells that possess increased responsiveness to m157 (Dokun et al., 2001; Bubic et al., 2004; Sun et al., 2009). This was an example of NK cell memory that was defined by specific NK cell receptor recognition of viral antigens. Additional adoptive transfer studies in mice have revealed that liver-resident NK cells have responded to several other pathogens including HSV-2 (Abdul-Careem et al., 2012), Vaccinia virus (Gillard et al., 2011) and Influenza A (IAV) (Li et al., 2017). Both the hapten and MCMV murine models demonstrated the specific recognition of foreign antigens by NK cells that contributed to a memory-like recall response but did not demonstrate if this occurred in humans and the extent NK cell memory contributed to virus control.

**Figure 1:**
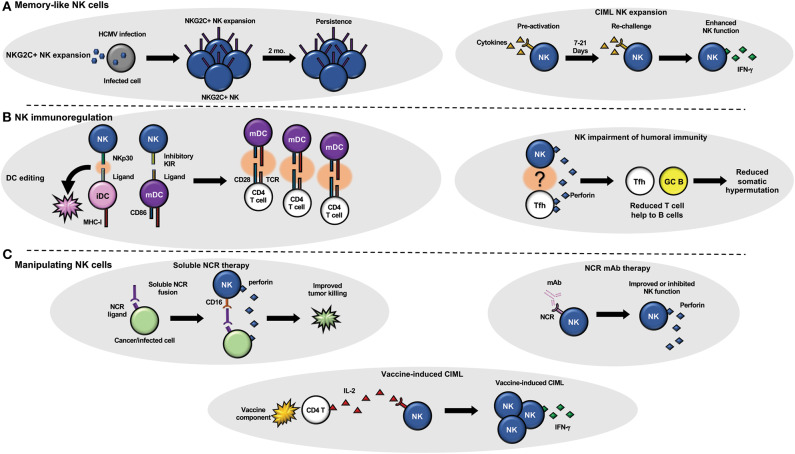
NK cells and adaptive immunity. **(A)** NK cells can develop memory-like attributes in response to infection (left) or cytokine stimulation (right). **(B)** NK cells have been shown to regulate adaptive immunity by targeting dendritic cells (left) that can change the quality of the T cell response and T follicular helper cells (right) that can regulate B cell maturation (somatic hypermutation) and function. **(C)** Therapeutic manipulation of NK cell function using soluble NK cell receptors that target tumor or viral infected cells to improve NK cell targeting (top left) or monoclonal antibodies that block or stimulate NK cell receptors (top right) to modulate NK cell function are under current development. Vaccine components such as adjuvants (bottom) could also be utilized to generate CIML NK cells *in vivo* with a vaccine.

NK cell adaptive response can also be mounted by stress signals expressed by infected host cells (Figure 1A). In humans, hantavirus-infected endothelial cells have been shown to upregulate HLA-E, a ligand for NK cell activating receptor NKG2C, subsequently resulting in the expansion of NKG2C+ NK cells, and the persistence of this subset up to 2 months post infection (Bjorkstrom et al., 2011). Similarly, HCMV infection of peripheral blood cells and fibroblast cells elicits expansion of NKG2C+CD57+CD56dimCD16+ circulating NK cells in humans in acute infection models (Beziat et al., 2013; Newhook et al., 2017).

Simian-immunodeficiency virus (SIV) vaccination and infection models in rhesus macaques have provided evidence that hepatic and splenic NK cells had the capacity to specifically target and kill SIV Gag and Env-specific dendritic cells (DC), and that this killing was NKG2C-dependent (Reeves et al., 2015). Recently published data by Nikzad et al. demonstrated that human liver-resident NK cells in humanized BLT mice displayed antigen-specific killing *in vitro* against HIV Env-loaded DC's 14 days post vaccination with recombinant HIV Env (Nikzad et al., 2019). Moreover, they demonstrated that human NK cell memory is long-lived in humans. Individuals that had Varicella Zoster Virus (VSV) infection in their youth were injected with a VSV-STA vaccine and had a significantly higher percentage of degranulating NK cells localizing at the site of injection, compared to controls. Another study demonstrated NK cell memory in Hepatitis B virus infection and vaccination (Wijaya et al., 2020). These findings provide much-needed evidence that antigen-dependent memory NK cells may be induced in humans, and that NK cell memory might have the potential to persist decades after initial sensitization.

## Cytokine-Induced Memory-Like NK Cells

NK cells can undergo differentiation into memory-like effectors once exposed to various cytokines such as IL-12, IL-15, and IL-18 (Figure 1A). These cytokine-induced memory-like (CIML) NK cells display higher IFN-γ secretion upon re-challenge compared to their naïve counterparts, and has been demonstrated in both mice and humans (Cooper et al., 2009; Romee et al., 2012; Keppel et al., 2013; Berrien-Elliott et al., 2015). CIML NK cells may also be defined by up-regulation of CD25 (Leong et al., 2014), as well as complete demethylation of IFN-γ promoter regions and other epigenetic changes (Lee et al., 2015; Wiencke et al., 2016). Indeed, IFN-γ promoter region demethylation of NK cells is also observed in the expanding NKG2C+ NK cells of HCMV-infected individuals, independent of the presence cytokine treatment (Luetke-Eversloh et al., 2014; Schlums et al., 2015). This similarity might imply that CIML expansion and persistence might depend on HCMV infection and/or NKG2C+ expansion, and that CIML phenotypes can be evoked independent of cytokine treatment (Goodier et al., 2016). One key difference in HCMV-expanded NKG2C+ NK cells is that *in vitro* or vaccine-induced CIML NK cells have been associated with expansion of less differentiated NK cells. CIML NK cells have been a key player in recent developments in cancer immunotherapy and have shown enhanced killing against a variety of cancer cell lines *in vitro*, including leukemia and ovarian cancer (Romee et al., 2012, 2016; Uppendahl et al., 2019). More recently, Romee et al. demonstrated enhanced killing of leukemic targets after adoptive CIML transfer into patients with acute myeloid leukemia (AML) and have conducted a clinical trial evaluating the safety of ALT-803—an IL-15 super agonist complex that activates NK cell and CD8 T cell function—in patients with hematologic malignancies who had suffered a relapse post-Hematopoietic cell transplant (HCT) (Romee et al., 2016, 2018). Another ongoing clinical trial aims at evaluating the efficacy of adoptively transferred CIML NK cells in relapsed AML patients after HCT (NCT03068819). Future studies optimizing the *ex-vivo* generation of CIML NK cells for immunotherapy of cancer as well as determining if CIML NK cells can be generated *in vivo* through a vaccine, adjuvant, or other cytokine-stimulating molecule will be necessary to further advance this area of research in the clinic.

## NK Cells Influence Adaptive Immunity Through Regulation of T and B Cells

NK cells and B cells have long been known to associate, given that NK cells mediate antibody-dependent cellular cytotoxicity (ADCC) through the NK cell Fc receptor, CD16. Recent evidence suggests that NK cells impact B cell affinity maturation and immune function (Figure 1B). Recent reports by Rydyznski et al. have elucidated that murine NK cells impair humoral immunity through the inhibition of CD4 T follicular helper (Tfh) and germinal center (GC) B cell expansion and function (Rydyznski and Waggoner, 2015; Rydyznski et al., 2015, 2018). Using an NP-KLH (4-hydroxy-3-nitrophenylacetyl; keyhole limpet hemocyanin) conjugate model for immunization in mice, they demonstrated that NK cell-depleted mice, compared to control mice, had higher Tfh and GC B cell populations, greater expansion of splenic germinal centers, and an increase in the production of NP-specific antibodies that displayed higher affinities for NP following immunization. NK cell impairment of B cell affinity maturation in mice was shown to occur in a perforin-dependent manner, as perforin-deficient mice displayed a similar level of affinity maturation as NK cell depleted mice did (Rydyznski et al., 2018). Other studies have shown that NK cells directly activate B cell IgG and IgM production, as well as facilitate immunoglobulin class-switching and can control HIV-1 neutralizing antibody responses (Snapper et al., 1994; Gao et al., 2008; Bradley et al., 2018). Conversely, NK cells have also been shown to have inhibitory roles in B cell function. Poly:IC injection in mice inhibited IgM primary response, via NK cell activation (Abruzzo and Rowley, 1983). T-cell dependent (IL-2) NK cell activation has also been shown to have negative outcomes for antibody production after EBV and pokeweed mitogen stimulation (Rydyznski and Waggoner, 2015). In human NK cell-B cell co-culture experiments, NK cells have been shown to activate B cell antibody production via TNFα (Becker et al., 1990) and CD40-CD40 ligand interactions (Blanca et al., 2001).

Studies in humans and mice have revealed that NK cells indirectly influence the T cell repertoire via direct interaction with antigen presenting cells, most notably immature Dendritic Cells (iDC) and mature Dendritic Cells (mDC; Figure 1B). Human DC-NK cell cross-talk and subsequent activation of both cell types was first reported *in vitro*, where it was reported that DC-NK cell interaction enhances NK cell activation and DC maturation, with the former expressing IFN-γ, stimulating the latter to mature, secrete IL-12, and amplify expression of the co-stimulatory molecule CD86 (Gerosa et al., 2002). In subsequent studies, mDC-derived IL-12 was shown to enhance CD8 T cell responsiveness and activation (Mocikat et al., 2003; Adams et al., 2005). The tendency for NK cells to kill iDCs while sparing mDCs, termed “DC editing” is another example of indirect changes to T cell immunity modulated by NK cells (Morandi et al., 2012; Ferlazzo and Moretta, 2014). The elimination of iDCs is hypothesized to enhance T cell priming, by decreasing competition between iDCs and mDCs which have the costimulatory molecules needed for T cell activation. In humans, this process is thought to be governed by ligand-receptor interactions of NKp30 ligands expressed on iDCs, and inhibitory KIR ligands expressed on mDCs (Ferlazzo et al., 2002).

During infection, NK cells target and kill infected host cells, which release antigen available for DC uptake. This enhancement of DC cross-presentation effectively improves cytotoxic T cell mediated immunity. After transfer of allogeneic B cells in mice, NK cell killing of the B cells resulted in apoptotic bodies taken up and presented by dendritic cells (Iyoda et al., 2002). NK cell killing of Ova-expressing splenocytes also resulted in release of antigen, leading to the enhancement of CD8 and CD4 T cell priming (Krebs et al., 2009). Activated murine NK cells are also capable of shaping T cell immunity directly. After activation, murine NK cells localize to the lymph nodes where they release IFN-γ, eliciting CD4+ T cell differentiation into the Th1 subtype (Martin-Fontecha et al., 2004).

## NK Cell Regulation of T Cell Immunity During Viral Infection

LCMV and MCMV infection studies in mice have produced variable results outlining the effect NK cells have on T cells during acute and chronic viral infection. Waggoner et al. demonstrated that NK cells targeted and killed CD4 T cells during LCMV infection in mice (Waggoner et al., 2011). However, other studies of LCMV infection have suggested that NK cells directly eliminated CD8 cells either through an NKG2D-dependent manner or another undefined mechanism during LCMV infection (Soderquest et al., 2011; Lang et al., 2012). Recently published data suggests that NK cells directly kill CD8 T cells during LCMV infection in mice, and that this killing is NCR-1 dependent (Pallmer et al., 2019). The presence of NK cells during LCMV infection in mice was reported to elicit T-cell exhaustion, and subsequently reduce both CD4 and CD8 T cell response to LCMV, and that NK cell depletion enhances T-cell mediated viral clearance (Cook and Whitmire, 2013). In chronic models of MCMV infection, TRAIL+ NK cells have been reported to target CD4 T cells in the salivary gland, which the authors suggest is to limit autoimmunity during chronic infection (Schuster et al., 2014). Other experiments showed that IL-10 secretion by NK cells during MCMV infection inhibited CD8 T cell response (Lee et al., 2009).

## Vaccinating for Memory NK Cell Generation

Vaccines have historically relied on eliciting antigen-specific effector and memory B and T cells to protect against subsequent infection, but for challenging pathogens such as HIV-1 and TB, alternative strategies to boost immunity must be pursued.

CIML NK cell induction during vaccination has a clear advantage over antigen-specific NK cell memory, as it is not restricted to certain antigens. CIML NK cells have been proven to be elicited after immunization with several human vaccines, including TIV, YF-17D, and BCG (Marquardt et al., 2015; Goodier et al., 2016; Suliman et al., 2016; Darboe et al., 2017). IL-15 has been demonstrated to prime TIV-vaccinated human PBMC to produce innate myeloid cytokines, as well as generate CIML NK cells that have enhanced responsiveness to H3N2 influenza virus (Wagstaffe et al., 2019b). The persistence and functional significance of vaccine induced CIML NK cells during vaccination requires further investigation.

Although the recent findings of antigen-specific human NK cell memory are useful, there is a dearth of literature outlining how human NK cells mediate antigen-specific killing as well as how long human NK cell memory can persist *in vivo*. Findings on VZV-specific NK cell memory was limited by the fact that VZV-naïve individuals are rare, and thus were not available to be used as controls in the Nikzad et al. study. The HCMV-induced differentiation of CD56dimNKG2C+ into adaptive like NK cells was shown to occur via an epigenetic mechanism, however, it is not clear if all disease models that display NKG2C+ NK cell expansion go through the same epigenetic changes that HCMV infection elicits.

It is hypothesized that some models of vaccine-dependent, antigen-specific memory NK cells occur through genomic rearrangement, rather than the epigenetic mechanisms displayed in HCMV infection/host stress signal models. Several studies have elucidated the correlation between antigen-specific CD4 T cell derived IL-2 and improved NK cell response in a number of different vaccination models (Figure 1C**)** (Horowitz et al., 2012; Jost et al., 2014; Goodier et al., 2016). Thus, the mechanisms of adaptive NK cell memory generation must be studied on a pathogen-dependent basis, if they are to be implicated in vaccination.

## Enhancing Classical NK Cell Effector Functions for Better Vaccination Outcomes

A roadblock inhibiting vaccine-induced NK cell effector function is the limited understanding of how these processes occur in humans, and how these processes vary across different human vaccine models. To date, much of the work concerning NK cell effector function has centered around IAV and HIV models in mice and humans. Prophylactic and therapeutic vaccine trials need to investigate key NK cell effector functions—namely PAMP and myeloid cytokine-induced NK cell activation, DC editing, and NK cell ADCC induction.

Pathogen-associated molecular patterns (PAMPs) are often incorporated as adjuvants in vaccines (Miyaji et al., 2011). PAMP-induced NK cell activation has been correlated with overall vaccine immunogenicity (Feng et al., 2013; Martins et al., 2014). A recent report suggested that the presence of IFN-g derived from PAMP-induced activated human NK cells amplifies the pro-inflammatory cytokine profile of dendritic cells (Oth et al., 2018). Although these findings need to be further investigated in specific infection and vaccination models, they suggest that the presence of PAMPs in conjunction with IL-2, enhance DC editing, and thus could be a contributing factor to the enhanced immunogenicity of PAMP-containing vaccines. Future efforts to determine which adjuvant or combinations of adjuvants that elicit superior NK cell function that results in improved protective adaptive immune responses should be considered for all vaccines to increase vaccine efficacy and durability.

Myeloid cell-derived cytokines (IL-12, IL-15, IL-18, IL-27) as well as T-cell derived IL-2 have all been documented to be involved with NK cell priming and activation. As described earlier, the IL-12, IL-15, IL-18 cytokine cocktail activated IFN-γ expression in NK cells during vaccination. A recent report has demonstrated that IL-27 promotes murine NK cell cytotoxicity and IFN-g production in an NKG2D-dependent manner during influenza infection (Kumar et al., 2019). Nanogram concentrations of IL-15 have been demonstrated to boost IL-12 production and boost human NK cell function after exposure to H3N2 *in vitro* (Wagstaffe et al., 2018). Many of these cytokines have been explored in terms of immunotherapy but have not been examined as extensively as adjuvants for vaccines against infectious disease. Influenza models would provide a convenient avenue to investigate the role that IL-27 plays in human models of influenza vaccination.

Improving NK cell ADCC via vaccination is also a key goal for Influenza, HIV-1, and other viruses (Hashimoto et al., 1983; Mielke et al., 2019). Recently, it was demonstrated that IL-15 is capable of improving ADCC-mediated killing against HIV-infected cells in seropositive donor plasma, and HVTN-100 vaccine trial (Fisher et al., 2019). In influenza-infected adults, ADCC antibodies specific to highly conserved viral proteins nucleoprotein (NP) and matrix 1 (M1) were found for both H1N1 and H5N2 strains of IAV (Vanderven et al., 2017). ADCC antibodies for M2, another highly conserved IAV protein, has been shown to elicit ADCC in mice, however human trials have not yet been conducted. Ebola-specific ADCC antibodies have been confirmed *in vitro* in human PBMCs and NK cell lines. Recently, it was reported that Ebola-specific ADCC is activated after various vaccination schedules of Adenovirus type 26.ZEBOV and modified vaccinia Ankara (MVA)–BN-Filo (Wagstaffe et al., 2019a).

Influenza and HIV remain persistent pathogens responsible for the deaths of millions every year. NK cell effector functions have been implicated in both disease models and should continue to be investigated. NK cells display direct recognition of influenza infected cells via interaction of NKp46 and hemagglutinin (HA). In mice, NK cells have been observed to localize to the lymph nodes during primary response to influenza vaccination and have ultimately been observed to regulate antibody production in an NK cell IFN-g and DC IL-6 dependent manner (Garcia et al., 2012; Chatziandreou et al., 2017; Farsakoglu et al., 2019). The exact mechanism behind the infection-induced IL-6 response remains unclear and should be investigated further in mice. The mechanisms by which NK cells regulate humoral immunity during influenza vaccination should be examined in mice and humans in order to investigate if an influenza vaccine with appropriate adjuvant combination can elicit a similar immune response as seen in the murine infection model. The positive effect that murine NK cells display during acute viral infection is interesting, given that chronic HIV infection models have suggested that functional NK cells play an inhibitory role in humoral immunity both in mice and humans—namely through inhibition of B cell maturation and decreased HIV-1 broadly neutralizing antibody production (Rydyznski et al., 2015, 2018; Bradley et al., 2018). In addition to cytokines and adjuvants, the use of soluble NK cell receptors and antibodies targeting activating or inhibitory NK cell receptors could be utilized to modulate NK cell function and influence the adaptive immune response during infection, autoimmunity and cancer (Figure 1C). The role that NK cells play in humoral immunity are dependent on the nature of an infection (acute vs. chronic) and the types of adaptive immune responses that are required to be protective.

## Closing Remarks

Here, we have summarized how NK cell phenotype and function can be manipulated to improve immunity to vaccines and cancer, and how NK cells can influence other arms of innate and adaptive immunity. Future efforts should attempt to elicit memory-like NK cell phenotypes, while enhancing innate NK cell effector functions. Limiting NK cell phenotypes that negatively impact the generation of protective immune responses must also be pursued. It should also be noted that future vaccination efforts should not seek to replace aspects of cell-mediated and humoral immunity with NK cell-mediated immunity but seek to modulate NK cell function in tandem with adaptive immunity.

## Author Contributions

SP, EG, and TB wrote and edited the manuscript.

## Conflict of Interest

The authors declare that the research was conducted in the absence of any commercial or financial relationships that could be construed as a potential conflict of interest.
